# Injections of Ether in Neuralgia

**Published:** 1890-08-09

**Authors:** 


### INJECTIONS OF ETHER IN NEURALGIA.

Dr. Kumps strongly recommends subcutaneous injections of ether, or, on account of the volatility of pure ether, of a mixture of equal parts of ether and alcohol, in the treatment of neuralgic and "rheumatoid" pains. He has used it in sciatica, lumbago, neuralgia of the shoulder, and facial and rheumatic neuralgia and torticollis, and suggests its use in hepatic colic for which it has long been given internally. The quantity of the mixture [Hoffmann's solution] he employs is one grain (16 ♏.) and one injection generally suffices, though several may be required for a complete cure. The local pain and irritation it produces pass off very soon after the operation.

